# Exceptional Response to Olaparib and Pembrolizumab for Pancreatic Adenocarcinoma With Germline *BRCA1* Mutation and High Tumor Mutation Burden: Case Report and Literature Review

**DOI:** 10.1200/PO.21.00437

**Published:** 2022-01-27

**Authors:** Joanne Lundy, Owen McKay, Daniel Croagh, Vinod Ganju

**Affiliations:** ^1^ Centre for Innate Immunity and Infectious Diseases, Hudson Institute of Medical Research, Clayton, Victoria, Australia; ^2^ Department of Molecular Translational Science, Faculty of Medicine, Nursing and Health Sciences, Monash University, Clayton, Victoria, Australia; ^3^ Department of Surgery, Faculty of Medicine, Nursing and Health Sciences, Monash University, Clayton, Victoria, Australia; ^4^ Peninsula and Southeast Oncology, Frankston, Victoria, Australia; ^5^ Department of Gastroenterology and Hepatology, Monash Health, Clayton, Victoria, Australia; ^6^ Centre for Cancer Research, Hudson Institute of Medical Research, Clayton, Victoria, Australia

## Introduction

Pancreatic cancer (PC) is currently the seventh leading cause of cancer death worldwide, but is predicted to become the second leading cause of worldwide cancer death by 2030.^1^ More than 80% of patients present with locally advanced or metastatic disease,^2^ and the mainstay of treatment in this setting is systemic chemotherapy.^3^ Despite incremental advances in recent years, prognosis remains poor with a median 5-year survival rate of just 10%.^4^

Barriers to the implementation of precision medicine in PC include a heterogeneous molecular landscape with most actionable changes occurring at low individual frequencies across the population,^5,6^ difficulties in accessing and sequencing high-quality biopsy material in a timely fashion,^7^ and patient factors including a propensity for rapid clinical decline.^7^

Here, we present a case of metastatic pancreatic adenocarcinoma harboring a germline *BRCA1* mutation and a high tumor mutation burden (TMB), demonstrating an excellent response to initial platinum-based chemotherapy, followed by a complete radiologic response to maintenance immunotherapy and poly (ADP-ribose) polymerase (PARP) inhibition.

## Case Report

A 76-year-old man was diagnosed with metastatic PC after presenting with fatigue and weight loss, on a background history of an acute myocardial infarction 6 weeks before. The Eastern Oncology Cooperative Group (ECOG) performance status (PS) at presentation was 2. His family history was significant for PC, diagnosed in his father in his early 70s. There was no family history of breast, ovarian, or prostate cancer.

**FIG 1.**
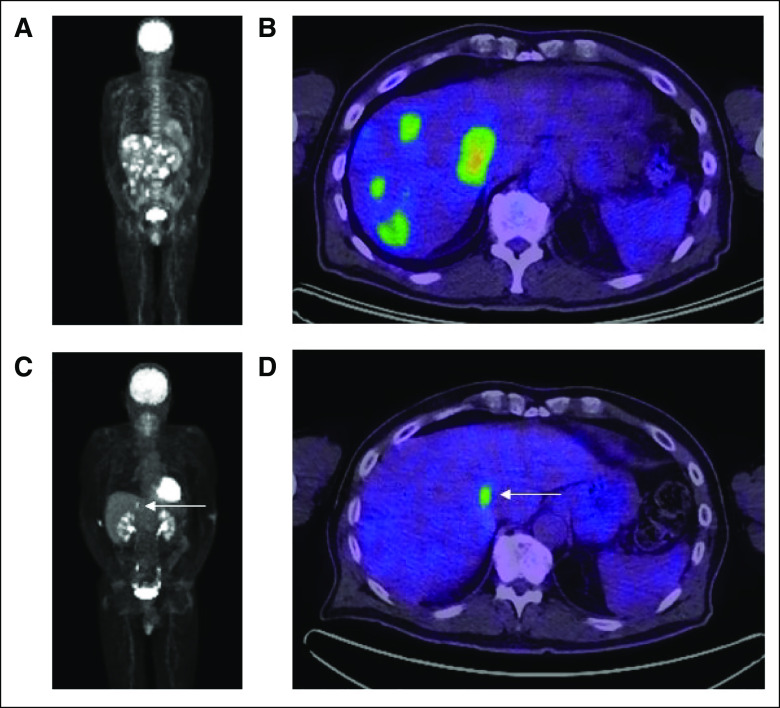
PET scan at baseline and after six cycles of carboplatin and nab-paclitaxel chemotherapy reveals excellent partial response to initial systemic therapy. Baseline PET scan including (A) MIP and (B) computed tomography fused axial views demonstrates pancreatic primary and extensive liver metastases. Follow-up (C) MIP and (D) fused axial views after six cycles of platinum-based chemotherapy with the addition of pembrolizumab from C4 demonstrate near-complete resolution of disease, with only a solitary remaining fluorodeoxyglucose-avid liver metastasis (arrow). MIP, maximum intensity projection; PET, positron emission tomography.

Baseline imaging revealed a 3 cm head of pancreas mass and diffuse extensive hepatic metastases (at least 20 lesions, with the largest measuring 48 mm; Figs 1A and 1B). Percutaneous biopsy of the liver was felt to be at high risk because of dual antiplatelet therapy, and he proceeded to endoscopic ultrasound, which confirmed a vascular head of pancreas mass and multiple liver metastases. An endoscopic ultrasound fine needle biopsy of a liver mass confirmed a diagnosis of metastatic poorly differentiated carcinoma. Immunohistochemical staining for cytokeratin 7 was positive, and mismatch repair (MMR) staining revealed a normal pattern of expression. Programmed death ligand-1 (PD-L1) status was not assessed.

Palliative chemotherapy was commenced with gemcitabine and nab-paclitaxel. The first cycle was complicated by a hospital admission for management of biliary sepsis, requiring a prolonged course of intravenous antibiotics, and severe recurrent upper gastrointestinal tract bleeding, requiring a total of eight units of packed red cells, four units of fresh frozen plasma, and two pools of platelets to stabilize. Ultimately, he also underwent angioembolization of the gastroduodenal artery and proximal branch of the superior mesenteric artery followed by palliative radiotherapy (20 Gy in five fractions) to control upper gastrointestinal bleeding. Subsequent clinical recovery was slow, with persistent fevers, fatigue, and a decline in PS. Because of these medical complications, there was a delay of 7 weeks between his first and second cycles of systemic therapy.

During this time, molecular analysis of his endoscopic ultrasound fine needle biopsy of liver was performed using the TruSight Oncology 500 (TSO-500) panel as part of the Endoscopic Ultrasound Molecular Evaluation of Pancreatic Cancer (EU-ME-PC) Trial (ACTRN12620000762954),^8^ and germline testing was arranged through a Familial Cancer Clinic using a targeted assay of 14 cancer predisposition genes. Both somatic and germline testing detected a pathogenic *BRCA1* mutation in exon 20 (c.5266dupC). The TSO-500 panel also detected pathogenic *KRAS* and *TP53* mutations (Table 1) and an extremely high TMB of 223.9 mutations per megabase (mut/Mb). There was no evidence of microsatellite instability.

**TABLE 1.**
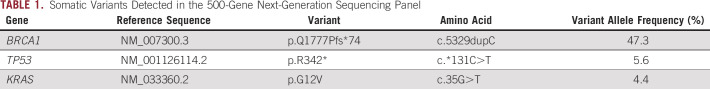
Somatic Variants Detected in the 500-Gene Next-Generation Sequencing Panel

These findings were reviewed in a local molecular tumor board, and on the basis of consensus recommendation, platinum-based chemotherapy was commenced (carboplatin and nab-paclitaxel). He completed six cycles of this regimen. In light of the extremely high TMB, he also elected to self-fund pembrolizumab, which was added from cycle four. He had an excellent clinical and radiologic response to platinum-based chemotherapy and immunotherapy (Figs 1C and 1D) and subsequently continued on maintenance pembrolizumab. His PS improved to ECOG 0. Because of persistent chemotherapy-induced anemia and in light of an excellent ongoing response to immunotherapy, maintenance PARP inhibition was considered but not commenced after completing chemotherapy.

**FIG 2.**
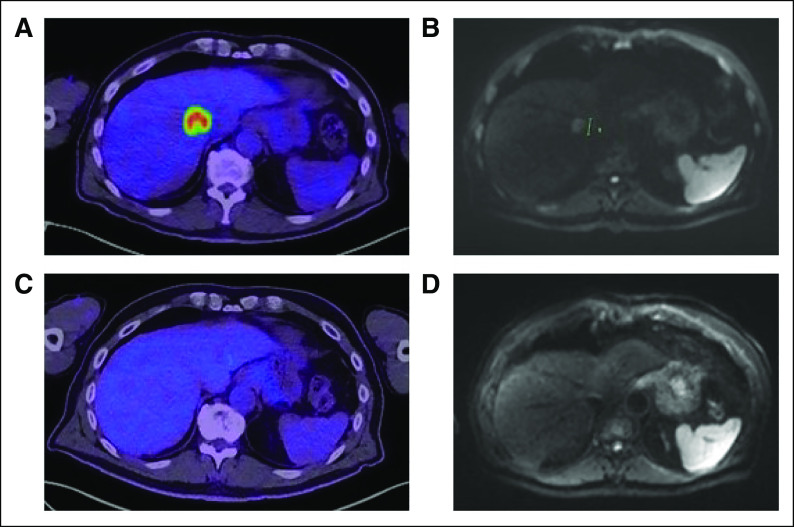
Response to olaparib after oligometastatic progression in liver. (A) PET scan and (B) MRI of liver after 5 months of maintenance pembrolizumab reveal progression in the sole remaining metastatic liver lesion. Olaparib was added, and 5 months later, a repeat (C) PET scan and (D) MRI demonstrate complete radiologic and metabolic response to therapy. MRI, magnetic resonance imaging PET, positron emission tomography.

Five months after commencing maintenance pembrolizumab (10 months after his initial diagnosis), imaging revealed an excellent ongoing response with near-complete resolution of the pancreatic and liver tumors. However, oligometastatic progression was evident in a solitary liver metastasis (Figs 2A and 2B), possibly representing a resistant clonal population. Because of the known *BRCA1* mutation, olaparib was added to the ongoing pembrolizumab therapy.

Ongoing therapy with olaparib and pembrolizumab has been well tolerated, and he remains clinically well with an excellent PS. Imaging 6 months after the addition of olaparib (16 months after his initial diagnosis) has revealed a complete radiologic response to therapy, with no evidence of residual active malignancy on positron emission tomography or computed tomography or magnetic resonance imaging (Figs 2C and 2D).

Clinical and molecular data for this patient were obtained from the Victorian Pancreatic Cancer Biobank and EU-ME-PC study databases after appropriate local institutional ethics board review (HREC/15/MonH/117 and HREC/61006/MonH-2020-200407). The patient described in this report provided informed written consent for the collection and publication of his clinical and molecular data and deidentified images.

## Discussion

Pancreatic ductal adenocarcinoma is typically diagnosed at an advanced stage, and systemic therapy remains the mainstay of treatment for this recalcitrant malignancy. Current ASCO and National Comprehensive Cancer Network guidelines recommend different regimens on the basis of PS. Patients with good PS (ECOG 0 or 1) are usually offered palliative chemotherapy with folinic acid, fluorouracil, irinotecan, and oxaliplatin or gemcitabine plus nab-paclitaxel, whereas those with poor PS (ECOG ≥ 2) are usually offered single-agent gemcitabine or best supportive care.^3,9^

Somatic driver mutations are common in pancreatic ductal adenocarcinoma and are dominated by *KRAS*, *P53*, *SMAD4*, and *CDKN2A*. Activating mutations in *KRAS* are detected in > 90% of patients with PC, with codon 12 mutations being most frequent. Until recently, attempts to target these pathways alone or in combination with other therapies have not yielded positive trials.^10^ However, the recent development of effective therapeutic targeting of *KRAS* G12C in advanced solid tumors^11^ raises hope for further therapeutic development targeting *KRAS*.

Targeted therapy approaches either alone (except for a few genomically defined subsets) or in combination with standard cytotoxic therapy thus far has overall proven to be disappointing in PC,^12^ with contributing factors likely including significant genomic heterogeneity, a complex tumor microenvironment, and a rapidly progressive disease phenotype. Despite these challenges, several recent studies have revealed that targeted molecular screening is feasible in PC^12-14^ and that patients who receive targeted molecular therapy may derive a survival benefit.^15^

Pathogenic germline mutations in *BRCA1* (as seen in this patient) or *BRCA2* and other related genes are seen in approximately 5%-9% of PCs.^16^ The recent demonstration of a survival benefit in patients harboring germline *BRCA* mutations treated with maintenance olaparib after platinum-based chemotherapy demonstrates the importance of identifying targetable molecular phenotypes in PC.^17^ However, further studies are required to determine the benefit of PARP inhibitors outside of the maintenance setting.

Microsatellite instability-high or mismatch repair–deficient (dMMR) tumors are rare in the PC population with a frequency of only approximately 1%-2% and are often associated with Lynch syndrome.^18^ Immune checkpoint inhibitors targeting programmed cell death protein 1 and PD-L1 are associated with improved survival in dMMR tumors.^19,20^ A recent systematic review identified that high TMB occurs in approximately 1% of PC and is commonly associated with dMMR status.^21^ High TMB is associated with immunotherapy response in other tumor types,^22,23^ but limited evidence to date does not reveal a clear correlation between high TMB and response to checkpoint inhibition in PC.^24-26^ Table 2 summarizes PC studies that include data on high-TMB patients.

**TABLE 2.**
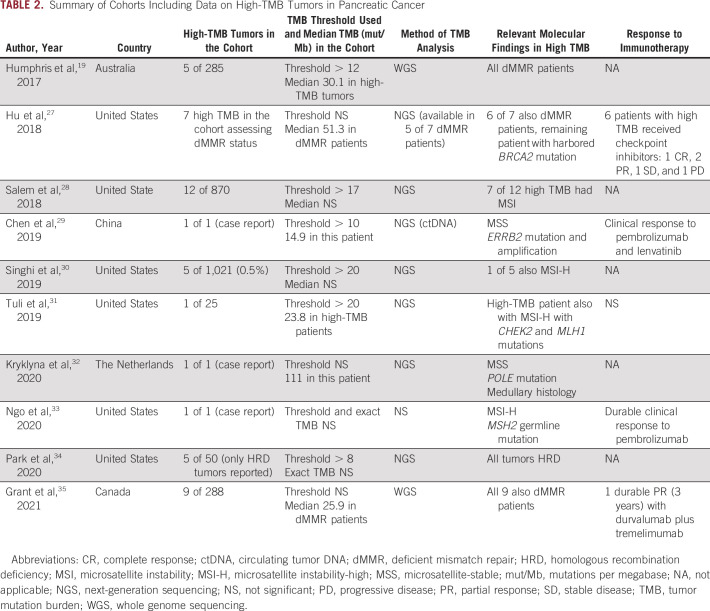
Summary of Cohorts Including Data on High-TMB Tumors in Pancreatic Cancer

This patient had an extremely high TMB and elected to self-fund pembrolizumab in addition to platinum-based chemotherapy, suggesting an immune-responsive tumor despite microsatellite stability. He demonstrated near-complete resolution of the pancreatic and liver tumors.

An updated understanding of PC pathobiology has prompted an exploration of potential therapeutic targets and heightened interest in implementing molecular sequencing into routine patient care. In addition to homologous repair deficiency genes, other molecular targets in PC may include *BRAF* V600E and *KRAS* G12C mutations, *HER2* amplification, *ALK* and *ROS1* translocations, and *NTRK* fusions.^12,13,36^ Novel immunotherapy has revolutionized the treatment of many cancers in recent years,^37^ but thus far has yielded relatively disappointing results in unselected patients with PC.^38,39^ Increasing evidence suggests that radiotherapy may enhance antitumor effects of immunotherapy through multiple mechanisms, and in our case, it is possible that radiotherapy acted as an immune primer.^40,41^ Further research is required to clarify the predictive utility of biomarkers such as microsatellite instability, PD-L1 expression, and TMB in PC.

In conclusion, this case report describes a profound clinical response to sequential platinum-based chemotherapy, pembrolizumab, and olaparib in a patient with advanced PC harboring a germline *BRCA1* mutation and high TMB. Although further studies are required to determine the role of TMB as a predictive biomarker for immunotherapy response in microsatellite-stable PC, this case suggests that remarkable responses can occur. In addition, although olaparib has been demonstrated to be an effective maintenance therapy in PCs with germline *BRCA* mutations, this case also alludes to a possible role for salvage therapy with olaparib in patients who progress on other therapies. To our knowledge, this is the first report of a complete response to combination therapy with pembrolizumab and olaparib after first-line platinum-based chemotherapy in PC. This patient's experience clearly demonstrates the immense potential benefits to be gained by implementing precision medicine in PC.
